# Supplementary material for: Are carbon emissions trading and green financial instruments synergistic? -Comprehensive quantitative research based on content analysis
Source: PLoS One. 2024 Mar 7;19(3):e0298601. doi: 10.1371/journal.pone.0298601 (PMC10919601; doi:10.1371/journal.pone.0298601)
Supplement: S1 Data — (ZIP) [file pone.0298601.s001.zip › Data/地级市Z_F工作报告.zip!/░▓╗╒/┬φ░░╔╜╩╨/└┤╫╘╣1⁄2╓┌║┼í╛┬φ┐╦╩2╛▌═°í┐_2015.docx]

# **政府工作报告（2015）**

【字体大小：[大](http://zwgk.mas.gov.cn/openness/detail/content/javascript:doZoom(18)) [中](http://zwgk.mas.gov.cn/openness/detail/content/javascript:doZoom(15)) [小](http://zwgk.mas.gov.cn/openness/detail/content/javascript:doZoom(14))】

各位代表：

　　现在，我代表市人民政府，向大会作工作报告，请予审议，并请市政协委员和其他列席人员提出意见。

　　一、2014年工作回顾

　　过去的一年，面对复杂严峻的宏观环境和艰巨繁重的工作任务，我们在省委、省政府和市委的正确领导下，在市人大、市政协的监督支持下，认真贯彻落实党的十八大、十八届三中、四中全会和习近平总书记系列重要讲话精神，以扎实开展党的群众路线教育实践活动为动力，围绕“项目突破年”活动部署要求，突出转型升级、加快发展主题，坚持稳中求进，沉着应对挑战，奋力攻坚克难，基本完成市十五届人大三次会议确定的目标任务。预计全市实现地区生产总值1320亿元，增长9.5%；财政收入202.7亿元，下降10.3%；规上工业增加值625亿元，增长11.8%；固定资产投资1670亿元，增长17%；社会消费品零售总额340亿元，增长13.2%；城镇居民人均可支配收入30950元，增长9.1%；农村居民人均可支配收入13920元，增长12%。

　　（一）经济发展稳中有进

　　工业经济平稳较快增长。制定出台工业经济倍增行动计划和四个配套政策，提出规上工业增加值和规上工业企业数四年倍增目标，明确重点产业发展方向，整合确定16个工业集中区作为下一步全市工业经济发展的重点区域。坚持领导带头招商，突出工业项目，改进考核方式，推行实地评估，招商引资工作取得新成效。全年实际利用省外亿元以上项目到位资金577亿元，外商直接投资16.6亿美元，均增长12%。工业项目新签约500个、新开工240个、竣工140个，新开工工业项目完成投资135亿元，均超额完成目标任务。标准化厂房新开工93万平方米、入驻企业209家。加大重点项目推进力度，山鹰80万吨造纸等115个重点项目基本建成，威博新能源等166个重点项目开工建设。深化与中央和省驻马单位合作，支持马钢转型发展，马钢收购法国瓦顿公司，云计算、煤焦油深加工等项目签约；十七冶集团晋升房建特级资质；中钢天源电子科技产业园一期建成投产；支持中钢矿院推动科技成果转化；与中冶华天合作组建节能环保研究院；安工大工商学院落户当涂县。深入持久开展服务企业活动，帮助企业协调解决7200多个问题；举办银企对接会、产学研对接会、产销对接会和银行行长进企业活动；中小微企业还贷应急周转金为33户企业垫付“过桥”资金10.6亿元；新成立国有担保公司4家，全市8家国有担保公司在保余额32.8亿元，放大2.8倍，这些举措对于提振市场信心、支持企业发展发挥了重要作用。全年完成“个转企”2741户，新增规上工业企业193家，总数达923家。

　　现代服务业集聚发展。预计完成服务业增加值400亿元，增长7%，占GDP比重超过30%，提高1个百分点。“一基地四园区”实施方案获国家发改委批复，钢晨物流园一期建成投产；和县“云谷”产业园、雨山区青年电商园等项目积极推进；软件园入驻企业150家、获批国家级科技企业孵化器；秀山金融街获批省级现代服务业集聚区；组建市信息化投资公司和工业设计中心。金融机构本外币贷款余额1103亿元，社会融资总规模437亿元，直接融资规模143亿元。举办中国（马鞍山）互联网大会、第三届海峡两岸（马鞍山）电博会等各类展会81场。万达广场、红星美凯龙建成开业，入选福布斯“中国大陆最佳商业百强城市”，被评为“中国电商百佳城市”。加快推进含山、和县现代农村商品流通服务体系试点县建设。农村“新网工程”建设取得新的成果。制定出台文化旅游发展行动计划，组建市文旅集团，积极推进大青山李白文化旅游区、濮塘运动健康休闲产业园等文旅项目，旅游业总收入达142亿元。入选全国养老服务业发展综合改革试点城市，三县均建成救助站、福利院，新增社会养老床位1634张。房地产业完成投资232亿元，商品房成交面积219万平方米。

　　（二）转型升级步伐加快

　　科技创新能力显著增强。获批省级创新型试点城市，创建国家知识产权示范城市培育期工作通过验收。全社会研发经费投入占GDP比重达2.25%，提高0.24个百分点。在全省率先实施科技创新券制度，共为271户企业发放创新券5655万元，撬动企业科技创新投入超2亿元。新增科技“小巨人”（培育）企业50家、高新技术企业50家、高新技术产品285个，实现高新技术产业产值780亿元。实现战略性新兴产业产值510亿元，增长12.5%。新增省级以上研发机构14家，组建皖江蔬菜产业技术研究院。授权发明专利320项，获省专利金奖3项。市经开区获批国家新型工业化产业示范基地，示范园区等4家园区获批省新型工业化产业示范基地，慈湖高新区跻身省首批知识产权示范培育园区。新认定省著名商标24件，“影星及图”荣获中国驰名商标。启动全国质量强市示范城市创建，被评为“中国年度质量魅力城市”。机器人产业区域集聚发展试点工作稳步推进。华菱西厨、三联泵业、学府信息进入“新三板”挂牌，5家企业进入省股权交易市场挂牌。推进专利权、商标权质押融资工作。制定产业投资资金管理办法，新设立产业基金2支。新增院士工作站2家、博士后工作站1家、国家级技能大师工作室1家，新引进高层次科技人才团队6家。

　　对外开放水平不断提高。郑蒲港一期工程开港运行，改变了安徽江北没有万吨级深水良港的历史。马鞍山港慈湖综合码头、天顺港集装箱堆场等项目开工建设，长江港口等3个企业码头完成2万吨级升级改造。综合保税区申建工作取得新进展，郑蒲港口岸扩大开放工作顺利推进，参与长三角通关一体化改革，推进关检“三个一”改革试点，外贸便利化水平不断提升，就地出口报关单增长1.8倍。制定鼓励港口和口岸发展政策，完成港口吞吐量8100万吨，增长8%；集装箱量10.5万标箱，增长50%。实现外贸进出口30亿美元，外经营业额3.6亿美元，对外投资增长2.5倍。进一步加大了与南京、芜湖的对接合作，宿马产业园连续两年获得省评估考核第一名。

　　（三）农业农村稳步发展

　　农业改革发展成效明显。围绕现代都市农业发展定位，通过组建产业联盟、专家顾问团、创业孵化器，促进新型经营主体逐步壮大、规模经营水平不断提高，新增各类新型经营主体413家，承包地流转比重达51.7%。农业产量稳步增长，完成粮食产量106万吨，增长4.4%；农产品加工业产值343亿元，增长22%。举办第八届和县蔬博会暨首届农业嘉年华等活动，全年乡村旅游游客量增长26%。扎实推进农村综合改革，土地确权登记颁证、集体资产股份制改革按计划推进。在全省率先实现农村产权交易平台全覆盖，完成11单土地流转交易，最高溢价率达54.7%。含山县被列入省级农村综合改革试点，在全省率先开展农村土地承包经营权抵押贷款风险补偿。与中信信托开展农村土地信托合作，流转土地承包经营权近2万亩。

美好乡村建设深入推进。22个省级中心村、33个市级示范村建设加快推进，当涂县被评为全省美好乡村建设先进县，含山县小冯村被评为全国美丽乡村建设示范村。改造县乡公路62公里、通村公路163公里、农村危桥24座、农村危房3353户。实施渔民上岸安居工程，安置渔民1554户。滁河防洪治理、慈湖河中游综合整治等水利重点工程顺利推进，完成45公里中小河流治理和14座小水库加固任务，解决了2.9万农村居民饮水安全问题。深入开展“三线三边”环境综合治理，扎实推进千万亩森林增长工程，新增造林面积10.3万亩。

　　（四）生态建设扎实推进

　　环境保护力度加大。制定出台生态市创建行动计划，完成各级生态建设总体规划修编，当涂县率先通过省级生态县验收。市经开区获批创建国家生态工业示范园区。完成省政府下达的220项大气污染治理工程目标任务，全年PM10平均浓度下降20%。扎实推进节能减排，含山光大生物质能发电等一批项目建成运行，万元GDP能耗下降4%；建成21项污染减排重点工程，主要污染物排放总量控制在省下达指标以内。水生态文明城市建设试点工作扎实推进。大力发展循环经济，通过全国再生资源体系建设试点市验收，慈湖高新区获批国家资源综合利用“双百工程”示范基地。生活垃圾焚烧发电项目完成招标。完成土地整治22.8万亩，获批建设用地2.3万亩、供地2.25万亩，工矿废弃地复垦利用试点成果获国土部肯定，和睦山铁矿被评为国家“绿色矿山”。进一步规范征迁拆迁工作，推动控违工作常态化。

　　城市功能逐步完善。宁安城际铁路马鞍山段、合福铁路含山段加快建设。205国道改造南段全面建成、北段稳步推进。226省道改造一期建成、二期开工。裕溪河大桥建成通车。马滁高速、206省道改造积极推进。九华路过江隧道、北沿江铁路等重大项目前期工作扎实推进。建成城区主干道路24公里，霍里山大道南延等5个项目完工。编制完成城市地下空间开发利用和地下综合管网规划。新建和续建18项电力重点工程，新增变电容量25万千伏安，供电保障能力得到提升。实现廉租房和公租房并轨，保障性住房新开工2.4万套、竣工9436套。新增城市绿道62公里、绿地462万平方米。开展架空线缆、施工扬尘、抛洒滴漏等专项整治活动，市容环境进一步改观。

　　（五）公共服务水平提高

　　民生实事顺利实施。33项民生工程投入资金34.4亿元，同比增长31%。新增城镇就业5.3万人，期末城镇登记失业率2.8%。五项社保新增参保2.8万人，成为“全民参保登记计划”全国试点城市。企业退休人员人均每月增加养老金168元，城乡低保、农村五保供养和孤儿基本生活费标准均提高10%。8件民生实事全面完成。重组市公交集团，公交服务保障能力得到提升；公共自行车租赁系统投入运行，日均使用近2万次；教育信息化工程快速推进，每7名学生拥有1台电脑，教师配备电脑率达66.7%；建成8个标准化县区急救分站，在全省率先实现120急救城乡一体化；高污染汽车限行管理系统投入使用；改造提升11个老旧小区；完成5家菜市场标准化改造；基本建成2个废旧物品分拣中心。

　　社会事业协调发展。全面启动教育综合改革，推进名校集团化办学，新改扩建公办幼儿园9所，新建农村标准化教学点50个，市二中郑蒲港分校建成投入使用。创新职业教育发展模式，成立市职教联盟，组建10个产教融合协作委员会。深化医改工作取得实效，在全省率先试点分级诊疗，基本建成市级区域卫生信息平台，县级公立医院全部实行药品零差率销售。秀山医院主体封顶，妇幼保健院晋升三级甲等医院，十七冶医院获批设置三级医院。稳妥有序实施“单独两孩”政策，低生育水平保持稳定。全力做好全国文明城市复评迎检工作，更加注重长效管理，推进创建工作常态化。成为全国10个公共文化服务标准化试点地区之一，市图书馆总分馆模式全国领先，市文化馆数字化改造列入文化部试点。建成35个乡镇综合文化站和7个农民文化乐园。节俭举办第26届李白诗歌节，荣获“中国诗歌之城”称号。群众体育蓬勃发展，荣获省第十三届运动会团体总分第二名。加强民生价格监管，物价水平保持稳定。市红十字会创新工作模式得到中国红十字总会肯定。新闻出版、广播电视、文学艺术、社会科学、档案史志工作水平不断提高。外事侨务、港澳台、妇女儿童、老龄、残疾人和慈善事业得到加强，邮政、气象、地震、防灾减灾工作扎实推进。

　　社会治理全面加强。推动社会治理重心下移，扁平化、网格化、信息化管理和社会化服务得到加强。完成村“两委”换届工作，推进村居干部职业化。创建“精品”、“四优”社区43个，新增各类社会组织95个，荣获“全国和谐社区建设示范城市”称号。坚持领导干部开门接访、重点约访、带案下访，开展信访积案化解专项活动，整合优化基层综治维稳信访平台资源，信访形势和秩序进一步好转。深入推进“平安马鞍山”建设，构建立体化数字化社会治安防控体系，开展重点工程保平安活动，严厉打击违法犯罪，社会大局保持稳定。公共法律服务体系建设全面启动，社区戒毒康复体系建设扎实推进，社区矫正、人民调解和法律援助工作积极推进。深化安全生产管理体制改革，列入全国安全隐患排查治理体系建设试点城市，安全生产形势稳定好转。持续加强食品药品安全监管，全力保障人民群众食品药品安全。强化应急平台和专业队伍建设，有效处置各类突发事件。民族团结、宗教和睦局面进一步巩固。国防教育、国防动员、人民武装、人民防空、双拥优抚工作得到加强。驻马部队和广大民兵预备役人员在促进社会稳定、支持地方建设等方面作出重要贡献。

　　（六）政府建设不断加强

****重点领域改革有序推进。****深化行政审批制度改革，取消、下放、调整市级行政审批事项达49.5%。推进企业注册登记制度改革，在全省率先开展“四证（照）一体”登记服务；实行园区为入园企业全程代办审批事项，提高投资项目审批服务效率；推行基层为民服务全程代理，政务服务标准化建设成效显著。推进政府职能转变和机构改革，建立市级政府权力清单、责任清单、涉企收费清单，完成工商、质监、食品药品监管体制改革，调整完善秀山新区、滨江新区管理体制，深化广电台内部机制改革，完善公共资源交易监管机制，落实事业单位分类和登记管理改革任务。

****依法行政能力得到提升。****认真执行市人大及其常委会决议、决定，自觉接受市人大法律监督、工作监督和市政协民主监督，认真办理市人大议案2件，建议、批评、意见106件，市政协提案287件。完善政府部门与各民主党派、工商联、无党派人士对口联系制度，进一步加强协商民主建设。密切与工会、共青团、妇联等人民团体联系。认真开展规范性文件合法性审查和备案工作，办理行政复议案件187件。坚持市政府常务会议集中学法，进一步规范市政府系统重大事项决策行为，严格实施法制监督，法治政府建设得到加强。

****作风效能建设常抓不懈。****扎实开展党的群众路线教育实践活动，切实解决群众反映强烈的突出问题，清理办公用房14.2万平方米，“三公经费”下降25.1%。深入开展“走亲戚”活动，干群关系进一步密切。实行政府及各部门主要负责人会议末位表态制度和不直接分管财务、项目等具体事务，强化对权力运行的监督制约。全面开展政策落实督查，推动政策“落地”、发挥实效。完善目标管理绩效考核，开展机关效能点评，推行工作日志制度，促进各项工作提质提效。加强重点领域、关键环节的行政监察和审计监督，落实党风廉政建设主体责任，全面推进廉政风险防控，树立政府为民务实清廉的良好形象。

　　各位代表！

　　过去一年的成绩来之不易。这是在省委、省政府和市委的坚强领导下，在国内经济总体下行、资源型城市转型的双重压力下，全市人民奋发进取、团结拼搏的结果。在此，我代表市人民政府，向辛勤工作在全市各条战线上的广大干部群众，向市人大代表、市政协委员、离退休老同志，向市各民主党派、工商联、无党派人士、人民团体和各界人士，向中央和省驻马单位、人民解放军、武警官兵和人民警察，向所有关心、支持、参与马鞍山建设发展的境内外投资者、海内外朋友们，表示衷心的感谢和崇高的敬意！

　　回顾总结去年的工作，需要特别报告的是，财政收入虽然完成了经市人大常委会批准调整后的目标，但降幅较大。究其原因，主要是受宏观环境影响，钢铁和与之相关的钢贸、废钢回收以及海关税收下降，房地产税收下降，非税收入下降，新增税源点不多。同时，我们也清醒地认识到，我市经济社会发展中还存在不少困难和问题。主要是：经济体量不大、结构不优，发展不充分、不平衡，传统产业比重较大、新兴产业规模偏小；服务业发展相对滞后，文旅资源没有得到充分开发，新的消费热点不多；投资增速持续回落，房地产市场仍处于调整阶段，部分行业和企业生产经营困难增多；部分县区政府负债较重，资金统筹难度加大；公共服务体系不尽完善，民生工作与群众期盼还有差距；资源环境承载压力日益加重，发展环境有待进一步提升。对此，我们一定高度重视，切实加以解决，决不辜负全市人民的期望和重托！

　　二、2015年工作安排

　　今年是“十二五”规划的收官之年，是全面深化改革、谋划“十三五”发展的关键一年，也是全面推进依法治市的开局之年，做好今年政府各项工作，任务艰巨、意义重大。当前，全球经济仍处在深度调整期，我国经济发展进入新常态，增长速度、发展方式、经济结构、发展动力都出现了新变化，对我们转方式调结构、培育新增长点、提高经济发展质量和效益都提出了新的更高要求。但总体上看，我国仍处于可以大有作为的重要战略机遇期的判断没有改变，宏观政策总体有利。特别是国家实施长江经济带战略，明确将安徽纳入长三角一体化发展规划，我市作为长江内河门户港，毗邻南京、合肥，战略地位凸显，产业基础雄厚，生态资源富集，市场空间广阔，发展潜力很大。我们要深刻认识新常态、主动适应新常态、积极引领新常态，顺势而为，乘势而上，在新的历史起点上奋力开创马鞍山转型升级、加快发展新局面。

　　今年政府工作的总体要求是：全面贯彻落实党的十八大、十八届三中、四中全会和中央、全省经济工作会议以及市委八届十三次全会精神，坚持稳中求进、进中求好、好中求快，坚持以提高经济发展质量和效益为中心，把转方式调结构放到更加重要位置，主动适应经济发展新常态，积极抢抓长江经济带建设新机遇，加快项目建设，做大经济体量，狠抓改革攻坚，突出创新驱动，强化风险防控，加强民生保障，促进经济持续健康发展、社会和谐稳定。

　　综合考虑，今年经济社会发展的主要预期目标为：地区生产总值增长8.5%，财政收入增长6%，固定资产投资增长13%，社会消费品零售总额增长12%，城镇、农村居民人均可支配收入分别增长7.5%和10%，城镇登记失业率控制在4%以内，单位GDP能耗、主要污染物排放总量控制在省下达指标以内。

　　为实现上述目标，我们将重点做好八个方面的工作：

　　（一）着力推进新型工业化，提高经济发展质量效益

　　深入实施工业经济倍增行动计划，以信息化为引领，大力发展新技术、新产业、新模式、新业态，促进工业总量扩张和产业质态提升，打造长三角地区重要的先进制造业基地。

突出项目拉动，做大工业体量。抢抓新一轮产业转移和企业布局调整机遇，坚持领导带头招商，推进全产业链招商，聚焦工业项目，积极承接有就业、有税收、有技术、有市场、无污染的优质项目，力争5000万元以上工业项目新签约500个、新开工275个、竣工160个，新开工工业项目完成投资160亿元，续建项目完成投资240亿元。注重加强与国内外知名产业研究院、专业咨询公司以及各类基金机构合作，力求在招大引强中把握先机、赢得主动。推进标准化厂房建设，吸引中小微企业入驻。加强项目谋划，科学编制“十三五”规划，谋划推进一批牵动性强的重大项目。大力推进企业技术改造，鼓励有条件的企业以市场换投资、资源换资本方式，开展兼并重组、整合发展。全年力争完成技改投资300亿元，新增规上工业企业200家，实现规上工业增加值640亿元。

****突出科技创新，提升产业质态。****以争创国家创新型试点城市为抓手，大力推动以产业化创新为重点的全面创新。继续实施科技创新券制度，引导企业加大研发投入，深化产学研协同创新，支持重点企业牵头组建产业研究院和技术创新联盟，加快科技成果转化和产业化。实施科技“小巨人”企业培育和创新能力提升计划，力争培育科技“小巨人”企业50家、高新技术企业40家，实现高新技术产业产值800亿元。大力引进科技中介服务机构，完善创业孵化服务链条，推进专利权、商标权质押融资，探索开展科技保险工作，发挥产业投资资金引导作用，加强与各类风险投资基金合作，推动科技型中小微企业加快发展。积极寻求与国内外高层次科技人才团队合作，力争新引进5家团队。加快全国质量强市示范城市、全国知名品牌示范区、国家知识产权示范城市创建步伐。

****突出融合发展，培育新增长点。****充分运用工业化和信息化深度融合的核心技术，推动传统产业高端化、低碳化、智能化改造，加快推进技术、产品、管理和商业模式创新。做大做强战略性新兴产业和高成长性产业，注重运用创业投资、股权投资，撬动社会资本跟进，加快机器人产业集聚区建设，力争战略性新兴产业产值增长15%。推进“一基地四园区”建设，加快软件园、电商园、金融街、工业设计中心、现代物流园区、融资租赁平台、区域性营销总部等生产性服务业平台建设，促进产业链向中高端迈进。高度重视互联网经济，积极发展与现代制造业相结合的云计算、大数据、物联网、互联网金融等新业态。加大对智能制造、轨道交通装备、3D打印、再制造、安全等新产业的培育扶持力度，促进其成长壮大。

****突出优化环境，助推企业发展。****深入持久开展服务企业活动，做好政策解读，及时兑现政策，增强服务企业意识，提高服务企业能力。加强企业家队伍建设，完善“360行，行行出状元”综合服务平台，提升劳动者技能。推进市与马钢融合发展，支持马钢做强钢铁主业，做活非钢产业，深化改革创新，加快转型升级。加大金融支持实体经济力度，加快民营银行组建步伐，完善政策性担保体系，引导金融机构加大产品服务和创新力度，指导中小企业发行集合债券，支持企业抢抓资本市场改革机遇、加快发展直接融资。抓好项目用地保障，力争获批建设用地1.5万亩，供地2万亩，实施土地整治20万亩。全面落实支持民营经济发展政策措施，深入推进工商登记制度改革，进一步优化民营经济发展环境。

　　（二）着力推进农业现代化，加快转变农业发展方式

　　围绕绿色食品供应、生态涵养调节、休闲观光旅游三大功能，以工业的理念、市场的理念抓农业，向农业输入现代生产经营要素和经营模式，促进农业强、农村美、农民富。

不断提升农业产业化水平。推进现代都市农业产业布局，培育壮大优势特色产业，努力打造绿色食品供应中心。加大农产品加工业招商，着力培育引进一批龙头企业，加快推进50个农业重点项目建设，力争市级现代农业示范区完成投资25亿元，规上农产品加工业产值增长18%。积极争创国家农业科技园区，支持当涂县争创国家现代农业示范区。加强粮食仓储设施建设，推进主食产业化。探索农业“生态循环”模式，推行农业标准化清洁生产，大力节水、节肥、节药，扎实推进生态农业和放心粮油工程建设，坚持走产出高效、产品安全、资源节约、环境友好的现代农业发展道路。

****加快构建新型农业经营体系。****发展适度规模经营，重点鼓励发展种养大户、家庭农场、农民合作社，发挥农业产业联盟作用，推动新型农业经营主体深化合作、联合发展。探索建立新型职业农民培育体系，造就一支适应现代农业发展的高素质职业农民队伍。健全土地流转服务体系，开展土地预流转，完善风险评估和调解仲裁机制。加快农村金融创新，加大农业保险支持力度，推进农村信用体系建设，扩大农村土地承包经营权抵押、担保贷款试点。深化与科研院所合作，加强农业专家顾问团建设，推广农业物联网应用。

全面深化农村综合改革。深入推进含山县省级农村综合改革试点县建设。加快“整市推进”农村土地确权登记颁证步伐，力争在全省率先完成确权任务。稳妥开展农村集体资产股份制改革。完善县级农村产权交易平台建设，进一步扩大交易范围、完善交易功能。深化小型水利工程产权制度改革，鼓励引导社会资本特别是农业新型经营主体，投资建设运营小型农田水利和水土保持工程。

****持久推进美好乡村建设。****扎实推进51个省级中心村、33个市级示范村建设，不断扩大美好乡村建设受益面。加强农村基础设施建设，改造50公里县乡公路、150公里通村公路、11座农村危桥，建成15座农村污水处理设施。推进滁河、水阳江下游防洪治理等水利重点工程，实施八小“水利工程”，重视解决农村居民饮水安全问题。坚持政策引导、市场化运作，支持秸秆综合利用项目建设。开展“三线三边”环境综合整治，推进千万亩森林增长工程建设，实施乡村旅游提升工程，组织乡村旅游节庆活动，支持星级农家乐建设发展，打造乡村旅游精品线路，加大宣传推介力度，努力把村庄建设、环境整治和产业发展有机结合起来，实现农村长治久美。

　　（三）着力推进现代服务业发展，促进消费扩大和升级

　　强化消费拉动作用，加快培育新的消费增长点，不断满足个性化、多样化消费需求，促进服务业发展提速、比重提高、结构提升。

****加快商贸流通体系建设。****认真落实商业网点规划，推进东誉城、金鹰国际、居然之家等项目建设，丰富商品种类，提升商业档次，打造城市商业集聚区，培育特色商业街，促进人气集聚和业态繁荣。加快社区商业服务网点建设，形成15分钟便利消费圈；推进含山、和县现代农村商品流通服务体系试点县建设，加快农村“新网工程”建设，提高为民服务水平。

****大力促进信息消费发展。以推****进国家信息消费试点市建设为契机，发挥市信息化投资公司作用，加快宽带基础设施建设，推动无线城市网络覆盖，普及信息化知识，增强信息产品供给能力，扩大移动互联网信息消费。以互联网思维创新服务理念、商业模式和管理手段，寻求与知名电商平台合作，推进“淘马鞍山”等本地电商企业做大做强，促进传统商业线上线下融合发展，大力发展农村电商，探索推进跨境电商，充分发挥电子商务对转方式、调结构、惠民生的促进作用。

****不断培育服务消费热点。****扎实推进全国养老服务业综合改革试点城市建设，支持社会力量兴办养老、家政、医疗、健康等服务机构，满足群众多层次、个性化服务需求。加强对“中华老字号”、“安徽老字号”培育，开展“马鞍山名小吃”认定，繁荣城市夜间消费，加快餐饮住宿业转型发展。大力发展假日经济、会展经济，释放消费潜力，促进消费增长。引导合理住房需求，稳定住房消费。提升教育、文化、体育消费。鼓励新能源汽车、节能产品等绿色消费。

　　（四）着力推进开放型经济发展，融入长江经济带建设

　　坚持以港兴市发展战略，培育壮大一江两岸临港产业，全力推进安徽江海联运枢纽中心建设，努力把马鞍山口岸经济区建成外向型临港制造业基地和国家级物流进出口基地。

****大力发展现代临港经济。****坚持规划引领，深入研究长江经济带总体规划，进一步修改完善我市各类专项规划，整合港口岸线资源，有序推进开发建设。全力支持郑蒲港新区建设发展，坚持建、养、运、管并重的工作思路，加快集疏运体系建设，完善相关配套设施，创新企业运营机制，提升港口服务水平。加强对外开放平台建设，力争综合保税区和口岸扩大开放早日获批，积极申报肉类、粮食、花卉等国家进口指定口岸，着力打造高效、便捷通关环境。充分发挥港口优势，培育壮大临港物流、装备制造、电子信息、食品加工等主导产业，加快推进御香苑冷链保税物流加工园、总部基地等项目建设，努力把郑蒲港新区打造成长江经济带和沿江产业走廊上的一个亮点。

****推动园区创新转型发展。****加快国家级开发园区功能提升，瞄准更高定位，强化创新驱动，提升发展质量，努力在推动全市开发园区转型升级中发挥示范引领作用。加快省级开发园区壮大升级，做大经济体量，完善配套功能，推进产城融合，提高错位发展、特色发展、集聚发展水平。支持当涂经开区创建国家级经开区，支持有条件的园区创建省级开发园区。认真落实工业集中区布点规划，促进土地利用规划、城镇建设规划、产业发展规划有机衔接，实现布局集中、土地集约、产业集聚。做强一批特色“区中园”，打造承接服务外包、集聚特色产业、服务创业创新的专业化园区。

****不断提高对外开放水平****。把握“一带一路”和长江经济带建设战略机遇，充分发挥区位、资源、市场等组合优势，着力打造安徽开放型经济的新高地。加快实施“走出去”战略，鼓励马钢、华菱星马等企业开展跨境先进技术合作，支持十七冶、中冶华天、建诚国际等企业承揽境外工程，引导中小企业开拓新兴市场、扩大产品出口。加大自主出口品牌培育力度，提高出口产品附加值和竞争力。支持企业扩大先进技术设备、关键零部件和战略性资源等产品进口。扩大对台经贸交流合作，认真研究对接上海自贸区，全方位融入长三角，深化与南京、合肥、芜湖对接合作。

　　（五）着力推进生态市创建，提升新型城镇化建设水平

　　坚持在保护中发展，在发展中保护，顺应人民群众对良好生态环境的新期待，大力推进绿色低碳循环发展，着力打造山清水秀、天蓝地绿的美丽家园。

****深入推进生态市创建。****坚持项目带动，重点实施十大生态建设项目。加大对基层创建指导力度，完成省级生态县区年度创建任务。持续打好大气污染防治攻坚战，深入开展工业废气、城市扬尘、机动车尾气、秸秆禁烧等专项整治，确保PM10年均浓度控制在省下达目标内。加大城市河道水系整治力度，推进集中式饮用水水源地保护，新建污水处理配套管网50公里。切实加强环境监管执法，坚决查处各类环境违法行为。加大节能减排力度，推行合同能源管理，加强节能评估与审查工作，确保实现能源消费强度和总量双控目标。大力发展风能、太阳能、生物质能等新能源和可再生能源，扩大天然气利用规模。大力发展节能环保产业，推行环境污染第三方治理，加快形成一批具有自主知识产权的节能环保装备和产品。积极推进生活垃圾焚烧发电、餐厨垃圾回收利用项目建设。加快市经开区创建国家生态工业示范园区步伐。落实最严格的水资源管理制度，实施阶梯水价，推进节约用水。完善节约集约用地制度和评价体系，开展批而未供和闲置土地新一轮清理工作。进一步规范矿产资源开采秩序，推动生态矿区建设。

****加快重大基础设施建设。****围绕打造江海联运枢纽中心，加快郑蒲港二期项目申报和北沿江铁路一期项目前期工作，推进马鞍山港慈湖综合码头建设。围绕推动一江两岸协调发展，积极推进合福铁路含山段、商合杭铁路含山段、马滁高速、巢无高速建设，加快推进226省道二期、206省道改造工程，开工建设367省道，做好九华路过江隧道项目前期工作。围绕加快与南京都市圈深度融合发展，支持宁安城际铁路马鞍山段加快建设，积极推进205国道北段改造、马濮旅游大道对接苏340省道、314省道对接苏341省道等项目建设，把轨道交通1号线规划建设作为宁马深度融合的重点工程，力争前期工作取得新进展。

****着力提升城市功能品质****。坚持“组团式、网格化、田园城市”总体思路，全面参与长三角城市群规划建设。强化“规划即法”意识，推进城市总体规划修编，做好控规编制及动态维护。开展城市设计，保护特色要素，加强规划管控，严格控制城市规模，促进城市建设与自然山水相融合、与人文历史相呼应，打造独具魅力的宜业宜居宜游城市。争取发行城市基础设施建设、棚户区改造专项债券，采取PPP等模式引入社会资本参与基础设施建设和运营。推进丰收路、慈湖河路等道路建设改造，重视解决城区部分道路拥堵、停车难等问题。积极推进“海绵城市”建设，扎实开展城市地下管网普查，完成10公里老旧管网雨污分流改造。建设60公里城市绿道，新增绿化面积320万平方米。进一步理顺城管执法机制，推进城管力量下沉，发挥数字城管作用，提升城市管理网格化、扁平化、数字化水平。支持博望区建设发展，加快重点基础路网和公用配套设施建设，提升博望区综合承载能力。扎实开展县城规划建设管理提升行动，支持重点镇建设。

　　（六）着力推进文旅融合发展，加快建设文化强市

　　抢抓皖南国际文化旅游示范区建设机遇，深入实施文化旅游发展行动计划，加快重点项目建设，开展A级景区创建，着力打造面向长三角地区的运动健康休闲中心。

大力发展文化事业。大力弘扬和践行社会主义核心价值观，不断加强公民道德建设，广泛开展志愿服务，深入推动文明城市创建常态化、长效化。推进公共文化服务标准化、均等化建设，确保通过国家公共文化服务体系示范区复查。加强诚信体系建设，培育诚信文化。实施文化惠民工程，推进市文化馆等改扩建工程，办好李白诗歌节等群众文化活动。实施文艺创作“五个一工程”，推出一批精品力作。推动公共文化服务向农村倾斜，建成2个乡镇综合文化服务活动中心。加强文化遗产保护。

****不断壮大文旅产业。****以“运动、健康、休闲”为主题，以“积极、阳光、快乐”为主基调，采取政府主导、企业主体、市场运作的模式，发挥市文旅集团作用，加大招商引资力度，整合一江两岸资源，推动全市文旅产业大发展。坚持项目带动，重点推进十大文旅项目建设，尽快完善景点周边配套，串点成线、联动发展、创出品牌。促进文化与科技融合，发展创意设计、动漫游戏、会展广告等新型文化业态。推动传统媒体和新兴媒体融合发展。以创建国家级文化产业基地为抓手，积极培育和引进各类文化企业，支持重点文化企业加快发展。

****提升景区开发水平。****实施采石古镇改造，整合周边景点资源，完善旅游配套设施，力争通过3年时间努力，将采石风景区打造成为全市首家5A级旅游景区。力争年内建成4A级旅游景区3家。增加旅游景区的文化、科技、创意、运动、体验、互动等元素，打造一批精品旅游线路。加快旅游公共服务体系建设，完成三区旅游标识牌设置，建成自驾游服务中心，推行“智慧旅游一卡通”，加大“课本上的马鞍山”等旅游产品线上营销力度，不断提升我市旅游景区的吸引力和影响力。

　　（七）着力推进社会事业发展，促进社会和谐稳定

　　坚持守住底线、突出重点、完善制度、引导舆论的基本思路，以推进基本公共服务均等化为导向，持续加大民生投入，加强法治社会建设，确保人民安居乐业。

****继续办好民生实事。****在实施好省里民生工程基础上，重点做好10件民生实事，包括：继续实施老旧小区提升改造、公交服务升级、教育信息化工程，实施高层住宅二次供水设施改造、居民小区配电设施提升改造、高龄老人居家养老服务、淘汰黄标车及老旧车项目、红十字应急救护培训项目、残疾人康复托养工程、农村卫生室达标工程。通过干好一件件实事，让群众真正得到看得见、摸得着的实惠。组织实施“全民参保登记计划”，不断完善城乡社会保障体系。稳步提高城乡居民养老水平，推进退休人员社会化管理服务全覆盖。探索儿童福利、残疾人康复、流浪乞讨救助及慈善工作模式创新。统筹推进保障房建设，新建保障房1.2万套，竣工8000套。加大住房公积金扩面工作。继续保持物价水平总体稳定。

****积极推进就业创业。****实施就业优先战略和更加积极的就业政策，加强与各高校合作，完善服务体系，吸引更多高校毕业生来我市就业创业。做好农村转移劳动力、城镇就业困难人员等重点群体就业工作，动态消除“零就业”家庭。制定鼓励全民创业指导意见，实施青年创业计划，用好互联网创业新平台，加快“青年创业园”建设，开展创业创新大赛，营造浓厚的创业创新氛围，掀起“大众创业、万众创新”的新热潮。采取更加有效的人才政策，延揽留住更多领军人才和创新团队来我市发展，为催生新企业、新产业注入源动力。

加快发展社会事业。继续加大教育资源

****向农村倾斜，推进一江两岸教育协调均衡发展****。支持三县和博望区中小学标准化建设。积极适应考试招生制度改革，推进普通高中多样化、特色化办学。支持安工大工商学院建设。深化职业教育教学改革，推进校企深度融合，加强校际交流合作，整合职业教育资源，加快现代职业教育体系建设，培养一大批高素质劳动者和技能型人才，更好服务发展、促进就业。深化公立医院改革试点，推进县级公立医院改革，完善基层医改成果，提升区域卫生信息平台，加快构建城乡分级诊疗体系。推进秀山医院、妇幼保健院门诊楼等项目建设，引导社会资本投资兴办医疗机构。坚持计划生育基本国策，稳定适度低生育水平。大力开展全民健身运动，举办市第十一届运动会。夯实基层统计基础，做好统计方法制度改革。推进气象现代化工作。启动不动产统一登记工作。加快发展妇女儿童、红十字、残疾人、老龄事业，积极发展民族宗教、档案史志、防灾减灾等社会事业。

****不断创新社会治理****。坚持运用法治思维和法治方式解决矛盾和问题，进一步提升人民群众安全感和满意度。扎实推进法治社会建设，深入开展法治宣传教育，广泛开展群众性法治文化活动。推进多层次多领域依法治理，深化和谐社区建设，更加注重发挥社会组织在法治社会建设中的积极作用。推进村居干部职业化，探索村居干部职级管理。加强基层信访平台建设，推进网上受理信访制度，做好信访积案化解工作，引导和支持群众理性表达诉求、依法维护权益。加强行业性、专业性人民调解组织建设，完善仲裁制度，提高仲裁公信力。继续深入开展“平安马鞍山”建设，确保社会安定有序。严格食品药品安全监管，切实保障人民群众饮食用药安全。强化安全生产责任体系、隐患排查治理体系和应急救援保障体系建设，推动安全监管网格化、精细化、专业化发展。完善危机预防和管理体系，不断提高应对各类事件和问题的智能化水平。推进国防动员、国防后备力量和人民防空建设，启动新一轮全国双拥模范城创建工作，推动军民融合、军地一体协调发展。

　　（八）着力推进政府自身建设，打造人民满意政府

　　科学把握新常态、新要求，依法全面履行政府职能，加快建设职能科学、权责法定、执法严明、公开公正、廉洁高效、守法诚信的法治政府。

****深入推进重要领域改革****。继续深化行政管理体制改革，加大简政放权力度，清理和简化前置审批条件，提高行政效能和服务水平。抓好市级政府权力清单、责任清单和涉企收费清单制度运行，加强动态管理和事中事后监管。全面建立和推行县级政府权力清单和责任清单制度，健全完善政府权力运行体系。继续稳妥推进事业单位分类改革。推动公务用车制度改革。实行全面规范、公开透明的预算管理制度，完善全口径预算体系，加强预算绩效评价，盘活财政存量资金，提高资金使用效率。进一步加强政府债务管理，规范新增债务举借，切实防范和化解债务风险。创新重点领域投融资管理机制，完善工程建设“六分开”体制机制，深化公共资源交易管理体制改革。完善江东控股集团管理体制和法人治理结构，做大做强业务板块，打造国有资本投资控股集团，带动全市投融资平台转型发展。完善开发区管理体制，强化产业支撑，推进产城一体化发展。

****提高依法行政工作能力****。牢固树立法治意识，自觉运用法治思维和法治方式履行职责，大力推动学法、尊法、守法、用法，把政府工作全面纳入法治轨道。进一步健全依法决策机制，规范政府系统重大行政决策行为，完善政府重大决策合法性审查制度，加强政策执行的跟踪督查和责任追究。积极争取地方立法权，做好前期准备工作。坚持严格规范公正文明执法，探索在开发园区和乡镇开展综合执法，健全完善行政裁量权基准制度。强化对权力运行的监督制约，自觉接受市人大法律监督、工作监督和市政协民主监督，依法执行市人大决议决定，认真办理市人大议案、建议和市政协提案，重大决策出台前向市人大报告、向市政协通报。完善政府部门与各民主党派、工商联和无党派人士对口联系制度，深入推进协商民主。自觉接受司法监督，高度重视舆论监督和社会监督，充分发挥工会、共青团、妇联等人民团体的重要作用。大力推进行政权力运行、财政预算、公共资源配置、公共安全等领域政府信息公开，让权力在阳光下运行。认真抓好政府系统党建工作，巩固党的群众路线教育实践活动成果，严格执行“八项规定”和省市有关规定，严控“三公经费”支出，推动作风建设常态化、长效化。严格落实党风廉政建设主体责任，深入推进惩治和预防腐败体系建设，坚持用制度管权管事管人，切实加强重点领域和关键环节监管，严肃查处各类违纪违法案件。

****保持奋发有为精神状态****。牢固树立进取意识，坚持解放思想、开拓创新，既要脚踏实地、埋头苦干，又要仰望天空、着眼未来，抢抓发展先机，争取战略主动，不断培育马鞍山发展新优势。牢固树立责任意识，把推动马鞍山转型升级、加快发展作为第一责任，扑下身子抓落实，心无旁骛抓发展，夙夜在公、勤勉工作，敢于担当、勇于负责。盯紧目标干，围着项目转，充分发扬“钉钉子”精神，一件事情一件事情去做，一个项目一个项目去推，一个问题一个问题去解决，坚持不懈，锲而不舍，水滴石穿，久久为功。牢固树立服务意识，始终坚持问题导向，认真解决群众反映的问题，满腔热忱地为基层、为企业、为群众办实事、办好事、办成事，努力让群众看到实实在在的成效。

　　各位代表！

　　实干收获希望，拼搏铸就未来。我们有决心、有信心担负起人民赋予的光荣使命。让我们在省委、省政府和市委的坚强领导下，同心同德，锐意进取，务实苦干，为开创马鞍山转型升级、加快发展新局面而努力奋斗！
